# A Generic Strategy to Create Mechanically Interlocked Nanocomposite/Hydrogel Hybrid Electrodes for Epidermal Electronics

**DOI:** 10.1007/s40820-023-01314-z

**Published:** 2024-01-12

**Authors:** Qian Wang, Yanyan Li, Yong Lin, Yuping Sun, Chong Bai, Haorun Guo, Ting Fang, Gaohua Hu, Yanqing Lu, Desheng Kong

**Affiliations:** 1 College of Engineering and Applied Sciences, National Laboratory of Solid State Microstructure, and Collaborative Innovation Center of Advanced Microstructures, Nanjing University, Nanjing, 210023 People’s Republic of China; 2 State Key Laboratory of Analytical Chemistry for Life Science, and Jiangsu Key Laboratory of Artificial Functional Materials, Nanjing University, Nanjing, 210023 People’s Republic of China; 3 College of Chemical Engineering and Technology, Engineering Research Center of Seawater Utilization Technology of Ministry of Education, State Key Laboratory of Reliability and Intelligence of Electrical Equipment, Hebei University of Technology, Tianjin, 300130 People’s Republic of China; 4 Key Laboratory of Intelligent Optical Sensing and Manipulation, Nanjing University, Nanjing, 210093 People’s Republic of China

**Keywords:** Stretchable electronics, Epidermal electronics, Silver nanowire, Conductive nanocomposites, Hydrogel

## Abstract

Stretchable electronics are crucial enablers for next-generation wearables intimately integrated into the human body. As the primary compliant conductors used in these devices, metallic nanostructure/elastomer composites often struggle to form conformal contact with the textured skin. Hybrid electrodes have been consequently developed based on conductive nanocomposite and soft hydrogels to establish seamless skin-device interfaces. However, chemical modifications are typically needed for reliable bonding, which can alter their original properties. To overcome this limitation, this study presents a facile fabrication approach for mechanically interlocked nanocomposite/hydrogel hybrid electrodes. In this physical process, soft microfoams are thermally laminated on silver nanowire nanocomposites as a porous interface, which forms an interpenetrating network with the hydrogel. The microfoam-enabled bonding strategy is generally compatible with various polymers. The resulting interlocked hybrids have a 28-fold improved interfacial toughness compared to directly stacked hybrids. These electrodes achieve firm attachment to the skin and low contact impedance using tissue-adhesive hydrogels. They have been successfully integrated into an epidermal sleeve to distinguish hand gestures by sensing muscle contractions. Interlocked nanocomposite/hydrogel hybrids reported here offer a promising platform to combine the benefits of both materials for epidermal devices and systems.

**Supplementary Information:**

The online version contains supplementary material available at 10.1007/s40820-023-01314-z.

## Highlights

- Nanocomposite/hydrogel hybrid electrodes are created with high interfacial toughness by introducing soft microfoams as the mechanically interlocking layer.
- In the hybrid electrodes, silver nanowires and hydrogels are electrically connected through the porous microfoams, achieving high conductivity and low contact impedance for high-quality biopotential recordings.
- The microfoam-enabled bonding strategy is generally applicable to diverse polymer substrates.

**Supplementary Information:**

The online version contains supplementary material available at 10.1007/s40820-023-01314-z.

## Graphical abstract

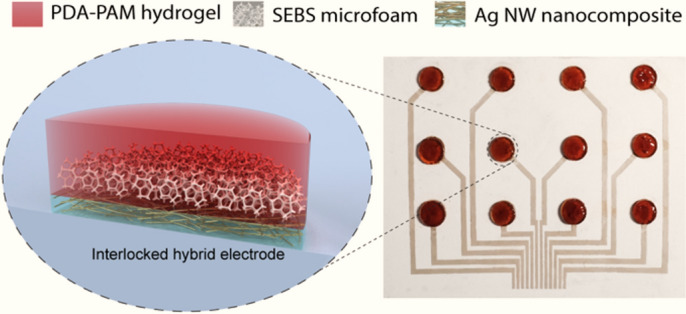

## Introduction

Stretchable electronics featuring compliant mechanical properties are disruptive innovations for next-generation wearables [1–3]. An attractive platform, called epidermal electronics, is established through the direct lamination of stretchable devices on the skin [4, 5], achieving robust body integration for emerging areas in health monitoring [6–8], wearable therapy [9], and human–machine interfaces [10, 11]. Compliant conductors are critical components of stretchable electronics to build functional electrodes and electrical interconnects. As an attractive class of material candidates, stretchable nanocomposites are formed by dispersing metallic nanostructures into elastomeric matrices, including carbon nanotubes [12, 13], metal nanoparticles [14, 15], metal nanowires [16–19], and metal nanoflakes [20, 21]. These nanofillers establish an electrically conductive and intrinsically stretchable percolation network. Despite the excellent electrical and mechanical properties, stretchable nanocomposites may not perfectly follow the textured skin with microwrinkles and hairs [22–24]. Air pockets developed at the tissue-device interface may affect the sensing performance by attenuating valuable biological signals [25]. In addition, stretchable nanocomposites lack the natural adhesion to biological tissues. These devices still require mechanical compression or bioadhesives to mount on the moving body [26–28]. These practical wearable issues largely limit the widespread implementation of stretchable electronic devices and systems.

Hydrogels are skin-like solids comprising crosslinked, water-filled polymeric networks. In biomedical fields, hydrogels have found a wide range of applications in contact lenses [29, 30], wound dressings [31–33], cell culture [34], and drug delivery [35, 36]. The ionic conductivity and mechanical stretchability render hydrogels attractive candidates for epidermal electronics [37, 38]. The extreme softness allows them to achieve conformal contact with the irregular surface of the skin [25]. In addition, the polymeric network can incorporate functional groups with cellular affinity for selective adhesion to biological tissues [39]. A stable and seamless interface is readily established by the tissue-adhesive hydrogel on highly textured skin, providing an ideal opportunity to collect physiological information and apply electrical/chemical simulations [40, 41]. Despite their excellent performance as epidermal interfaces, the limited conductivity of hydrogels from dissociated ions still restricts the expansion of application scopes toward various devices and systems.

A promising architecture of epidermal electronics involves heterogeneously integrated conductive nanocomposite and hydrogel hybrids to combine the best of both worlds. Directly bonding hydrogels to polymers is often challenging due to their distinctive surface energies [42]. As a straightforward approach, hydrogels and polymers are modified in bulk with silane coupling agents to achieve a strong bond at the interface [43]. The main drawback is the potential changes in the mechanical and electrical properties of nanocomposites when they are mixed with silanes [44]. Alternatively, the polymers are surface modified with reactive groups for hydrogels through oxygen plasma exposure [45], benzophenone-based photograph treatment [46, 47], and cyanoacrylate polymerizations [48]. The surface treatment may easily damage the conductive nanocomposites. In contrast to the molecular modifications, a macroscopically mechanical interlock strategy relies on geometric designs to achieve physical adhesion between polymers and hydrogels [49]. Although highly roughened interfaces are known for improved adhesion [50], this common surface engineering approach leads to small adhesion forces for hydrogels due to their inherent softness for easy separation through elastic deformations [42]. A porous elastomer web has been employed as the matrix to load conductive nanofilm electrodes and lock with the hydrogels [51]. This interpenetrating elastomer/hydrogel network can only be separated through rupture, which substantially increases the interfacial toughness [52]. Unfortunately, this attractive design relies on the porous microstructure of the substrates and cannot be directly applied to regular devices constructed on elastomer films. An effective approach to firmly combine conductive nanocomposite with hydrogels is therefore urgently required for epidermal devices.

In this study, we report a generic strategy to build mechanically interlocked conductive nanocomposite/hydrogel hybrid electrodes. Silver nanowire (Ag NW)/elastomer nanocomposites are spray deposited on the styrene-ethylene-butylene-styrene (SEBS) substrate to create patterned conductive features. A stretchable and porous SEBS microfoam is then thermally laminated to the Ag NW nanocomposite for mechanical interlock with the hydrogel. The resulting interlocked hybrid electrodes exhibited a high interfacial toughness of 158.2 J m^−2^, representing a notable improvement over directly stacked hybrids by a factor of 28. In addition to the SEBS substrate, a tackified microfoam is readily bonded with different substrates to construct robust hydrogel/polymer hybrids, including ethylene–vinyl acetate copolymer (EVA) elastomer, poly(methyl methacrylate)–poly(n-butyl acrylate) (PMMA-PnBA) elastomer, and polyethylene terephthalate (PET) plastic. After attaching the porous microfoam, the conductive nanocomposite still makes contact with the hydrogel through the porous microstructure, enabling interlocked hybrid electrodes to be electrically connected. The hybrid electrodes based on polydopamine–polyacrylamide (PDA-PAM) hydrogels exhibit conformal contact and strong adhesion to the skin, achieving low contact impedance better than state-of-the-art Ag/AgCl gel electrodes. The corresponding epidermal sensors can be mounted on the curvilinear body to record electrocardiogram (ECG) and electromyogram (EMG) signals without additional adhesive. Additionally, an integrated epidermal sleeve has been created composed of interlocked hybrid sensing electrodes and Ag NW nanocomposite electrical interconnects, functioning as a human–machine interface to distinguish different gestures by recording muscle contractions. The design and fabrication of mechanically interlocked hybrid electrodes are well-compatible with existing epidermal devices. The robust combination of conductive nanocomposites and hydrogels demonstrated here marks a significant step forward in the field of stretchable and wearable electronics.

## Experimental Section

### Materials

All thermoplastic elastomers are commercially available, including hydrogenated styrene–ethylene–butylene–styrene elastomer (SEBS, Tuftec H1221) from Asahi Kasei Corporation, poly (methyl methacrylate)–poly (n-butyl acrylate) (PMMA–PnBA, Kurarity LA2140e) from Kuraray Co., Ltd., and EVA (Elvax 40W) from DuPont de Nemours, Inc. All other chemical reagents were purchased from Shanghai Macklin Biochemical Co., Ltd. Ag NWs of ∼80 μm in length were synthesized in a polyol-reduction process [53]. Salicylic acid microrods were synthesized through an antisolvent crystallization process according to the reported formulation [54]. The polydopamine-polyacrylamide (PDA–PAM) was synthesized following a reported method with slight modifications [39]. Briefly, a mixture of 5 g acrylamide, 15 mg bisacrylamide, 500 mg ammonium persulfate, 20 mg dopamine, and 100 mg Aerosol OT-75 surfactant was thoroughly dissolved in 15 mL H_2_O under vigorous stirring inside an ice bath. After adding 60 μL tetramethylethylenediamine, the precursor solution was stirred for 2 min and then injected into a mold. The hydrogel was obtained after crosslinking at room temperature for 2 h. To prepare stretchable substrates, the thermoplastic elastomer pellets were dissolved in toluene and then drop cast onto non-sticky glass wafers with octadecyl trichlorosilane (OTS) functionalization, followed by thorough evaporation under ambient conditions.

### Preparation of Microfoams, Ag NW Nanocomposites, and Interlocked Hydrogel–Polymer Hybrids

#### Composite Films and Corresponding Microfoams

Salicylic acid microrods and a SEBS solution (20 w/w% in toluene) were mixed thoroughly in a laboratory blender (FS400-ST, Shanghai Lichen-BX Instrument Technology Co., Ltd.). The mass ratio between salicylic acid microrods and the SEBS elastomer varied and was typically set at 4:1. The viscous mixture was drop cast onto an OTS-modified glass wafer and dried under ambient conditions to obtain the composite film. The porous microfoam was formed by dissolving salicylic acid microrods in ethanol and then drying under ambient conditions. EVA-based composite films and corresponding microfoams were prepared in a similar procedure using EVA solution in toluene.

#### Patterned Ag NW Nanocomposites

As-synthesized Ag NWs were redispersed in chloroform at 1.0 mg mL^−1^. SEBS elastomer was thoroughly dissolved at 5.67 mg mL^−1^ in this Ag NW organic dispersion. The mixture was spray deposited into the conductive nanocomposite as described previously [53]. During deposition, stainless shadow masks defined the patterned features comprising sensing electrodes and electrical interconnects. An additional ∼20 μm-thick SEBS elastomer layer was spray deposited onto the interconnect regions for encapsulation. A flexible flat polyimide cable with copper wires was bonded to the interconnect ends as the interface with external recording equipment.

#### Mechanically Interlocked Hydrogel–Polymer Hybrids

Salicylic acid/SEBS composite films were thermally bonded to SEBS substrates in an automatic vacuum laminator (temperature = 80 °C, pressure = 70 kPa, and duration = 3 min). The embedded salicylic acid microrods were removed in an ethanol bath to produce the porous microstructure. Inside a mold, the hydrogel precursor was cast onto the microfoam-attached substrate. The anionic surfactant allowed the precursor to infiltrate the porous microstructure. The interlocked hydrogel–SEBS hybrid was obtained by curing in ambient conditions. To generalize the design for different substrates, a petroleum resin was added to the SEBS elastomer as the tackifier. A mixed solution was prepared by dissolving 2 g SEBS and 1 g petroleum resin in 12 g toluene. The resulting composite film exhibits firm adhesion to other substrates (Fig. S9). Interlocked hydrogel/polymer hybrids were prepared following similar procedures. Alternatively, salicylic acid/EVA composite films were created and thermally bonded with targeted polymer substrates, producing porous microfoams to interlock with the hydrogels.

### Material Characterizations

Optical microscopy images were collected with a Keyence VHX-6000 digital microscope. Scanning electron microscopy (SEM) images were acquired using a Zeiss GeminiSEM 500 field emission scanning electron microscope. Optical images were taken with a Fujifilm X-T10 camera. Optical topographical images were obtained using a Keyence VK-K1000 laser scanning microscope. The sample thickness was determined with the corresponding height profiles. All mechanical measurements were carried out using a Shimadzu AGS-X universal testing machine with a 50 N load cell. Uniaxial stress–strain curves were obtained from rectangular samples (20 mm length, 9.5 mm width, and 140 μm thickness) at a constant speed of 20 mm min^−1^. Interfacial toughness was determined from standard 180°-peeling tests at a constant speed of 50 mm min^−1^. All samples were cut into rectangular shapes with 20 mm in width and 80 mm in length. A 90 µm-thick polyimide film was glued to the hydrogel using cyanoacrylate adhesive as a stiff backing layer. An 80 µm-thick polyethylene terephthalate (PET) film was bonded to elastomer substrates as a stiff backing layer. The electrical resistance of conductive nanocomposites was measured under a four-probe configuration using a Keithley 2110 digital multimeter. All tensile strains were applied with a homemade motorized translational stage. Cyclic voltammetry was performed in 0.1 M PBS solution using a CHI 660E electrochemical workstation. A three-electrode setup was used with an Ag/AgCl reference electrode, a carbon rod counter electrode, and the working electrode.

### Impedance Measurement and Electrophysiological Signal Detection

A pair of electrodes were attached to the forearm. Skin–electrode contact impedance was acquired using the electrochemical workstation. Interlocking hybrid electrodes and commercial Ag/AgCl gel electrodes (2223CN, 3 M Co.) were measured separately for comparison. The EMG sensing patch was attached to the right flexor carpi radialis, whereas the ECG sensing patch was attached to the chest. All biopotential signals were conditioned with a preamplifier from Nanjing Zijin Electronics Studio and recorded at a rate of 2 kHz by a USB3202 data acquisition card from Beijing Art Technology Development Co., Ltd.

### Fabrication and Operation of Integrated Epidermal Sensing Sleeve

The integrated epidermal sensing system was composed of four channels of epidermal sensing electrodes. Each channel has three 1 cm electrodes spaced 2.7 cm apart. The epidermal sensing device was attached to the forearm with sensing electrodes along the key muscles. All channels were conditioned with an Intan RHD2000 amplifier and simultaneously sampled at 5 kHz with an Intan RHD USB interface board.

## Results and Discussion

### Design and Fabrication of Interlocked Hybrid Electrodes

**Fig. 1:**
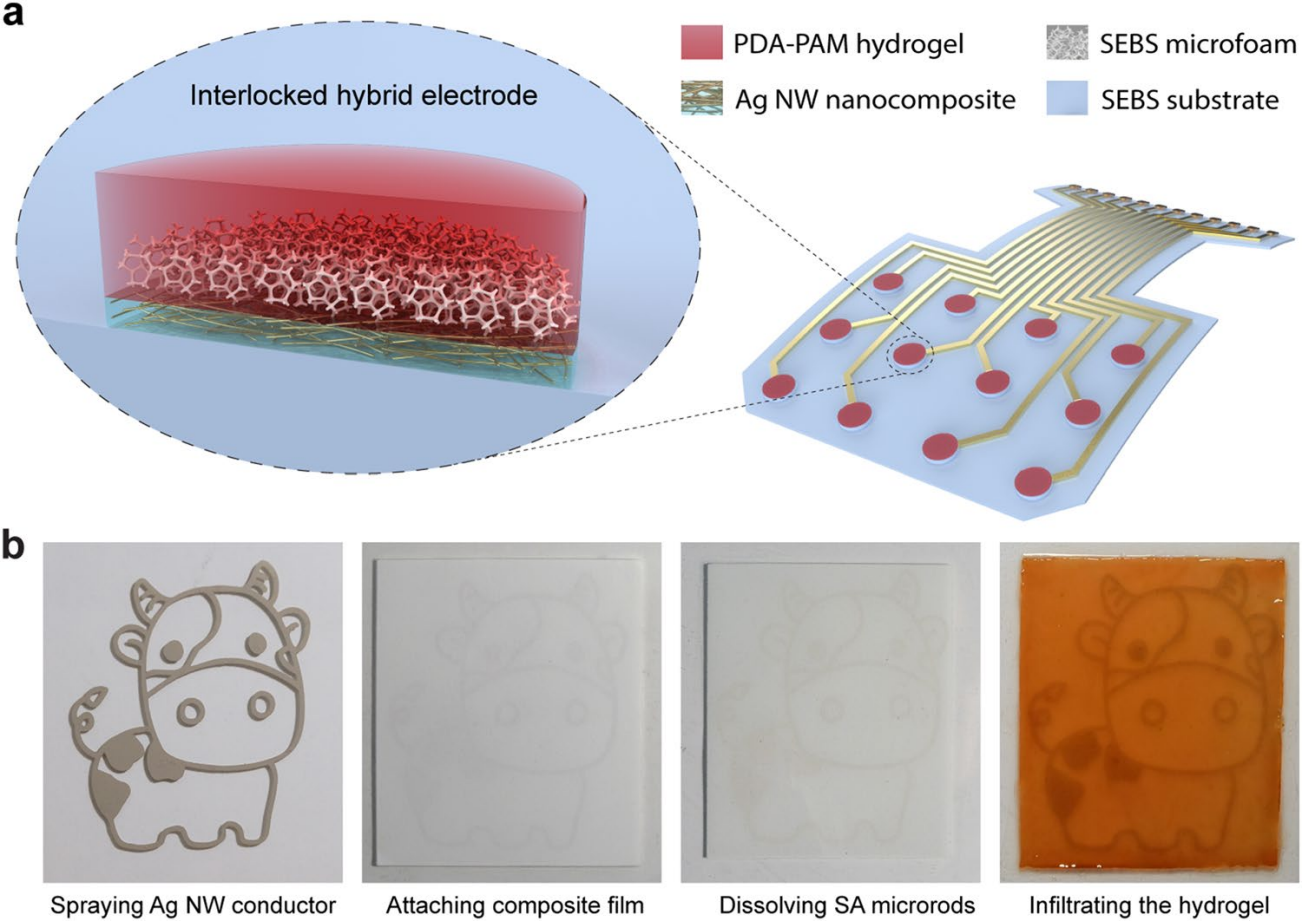
Design and fabrication of mechanically interlocked nanocomposite/hydrogel hybrid electrodes. **a** Schematic illustration of an epidermal sensing device comprising Ag NW nanocomposite/hydrogel hybrid electrodes and Ag NW nanocomposite interconnects. **b** Optical images of the process flow to create an interlocked elastomer/hydrogel hybrid involving spray depositing silver nanowire nanocomposites, attaching salicylic acid/elastomer film, dissolving embedded SA microrods, and infiltrating the hydrogel

As schematically illustrated in Fig. 1a, a representative epidermal electronic device is composed of Ag NW nanocomposite/hydrogel hybrid electrodes and Ag NW nanocomposite interconnects. Tissue-adhesive hydrogels are exploited in these hybrid electrodes to establish strong and conformal electronic interfaces with the skin. An interpenetrating hydrogel/polymer layer provides a robust interlocking mechanism for reliable attachment to the sensing device. Highly conductive Ag NW nanocomposites are used to construct electrical interconnects for external equipment. Figure 1b reveals the critical steps involved in preparing the polymer/hydrogel hybrids. Polyol-reduction synthesized Ag NWs are uniformly dispersed in a SEBS elastomer solution and spray deposited on the stretchable substrate as the conductive nanocomposite [53]. Conductive features are readily defined with shadow masks, as exemplified by the cow cartoon pattern. Soft microfoams are produced through a sacrificial template approach. Salicylic acid (SA) microrods through antisolvent synthesis are blended with SEBS elastomer to form a composite film (Fig. S1) [54, 55]. This composite film is physically attached to the conductive features in a vacuum laminator, in which the combined heat and pressure promote the interdiffusion of polymer chains for a strong bond [56, 57]. The embedded SA microrods are removed by dissolving ethanol to form the porous microstructure. The interlocked hybrids are realized by infiltrating the hydrogel precursor into porous microfoam and crosslinking under ambient conditions. Notice that mussel-inspired PDA–PAM hydrogel is adopted here for its excellent tissue affinity [39].

### Characterizations of the Bonding Mechanism

**Fig. 2:**
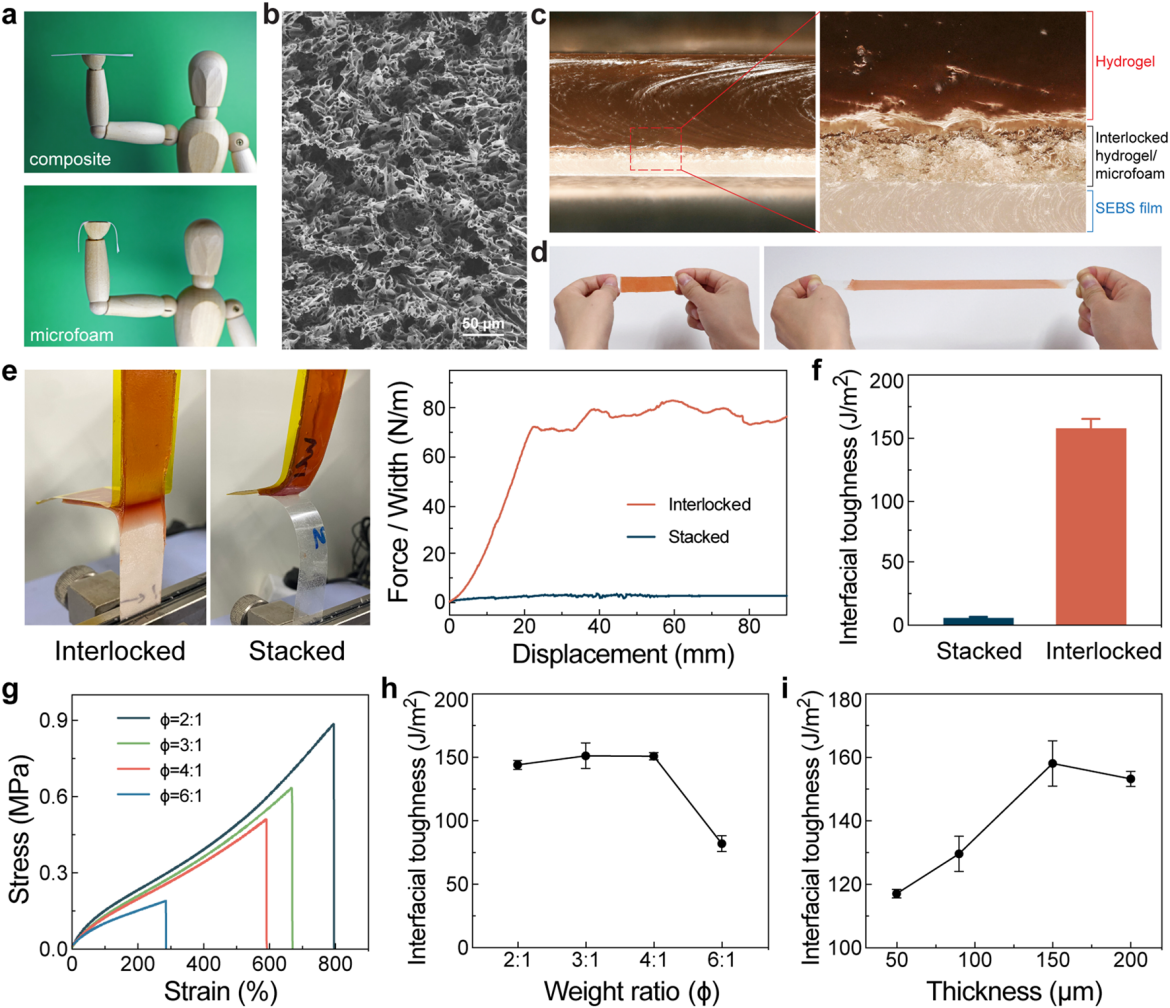
Mechanical characterizations of interlocked hybrid electrodes. **a** Optical images showing a self-supporting composite (top) and a soft SEBS microfoam (bottom). **b** SEM image of the microfoam with interconnected micropores. **c** Optical microscopy images revealing the cross-section of the interlocked hydrogel-elastomer hybrid. **d** Images of the interlocked hydrogel-elastomer hybrid under uniaxial tensile deformations. **e** 180°-peeling tests with optical images (left) and force *versus* displacement curves (right) for elastomer/hydrogel hybrids. Interlocked and stacked hybrids have been measured for comparison. **f** Corresponding interfacial toughness of the hybrids. **g** Uniaxial stress–strain curve of SEBS microfoam with different porosity, in which the porosity is modulated by the weight ratio (ϕ) between salicylic acid and SEBS elastomer. **h** Interfacial toughness as a function of microfoam porosity for ~ 150 μm-thick interlocked SEBS/hydrogel hybrids. **i** Interfacial toughness *versus* microfoam thickness (*ϕ* = 4:1)

The composite film is self-supporting and convenient to handle, as shown in Fig. 2a. In Fig. S2, the uniaxial stress–strain curve confirms its decent modulus of 4.73 MPa. In contrast, the SEBS microfoam is soft, porous, and easily deformable. The SEM image reveals the microstructure containing interconnected pores in dozens of micrometers (Fig. 2b). The microfoam has a low modulus of 0.15 MPa and a large fracture strain of 598% (Fig. S2). These distinctive mechanical properties are exploited to enable the physical bonding process. The composite film is stiff enough to facilitate the transfer and thermal lamination onto other substrates without any surface wrinkles. The porous microfoam is then formed by dissolving the embedded SA microrods. Strong attachment manifests with interfacial toughness of 1776.5 J m^−2^ (Fig. S3). In contrast, the soft microfoam is easily compressed and loses its porosity during thermal bonding, thereby eliminating the practical feasibility of direct transfer.

The PDA-PAM hydrogel has been synthesized by a free radical polymerization method. According to Fig. S4, the uniaxial stress–strain curve reveals its modulus of 0.54 kPa, fracture strain of 952%, and toughness of ~ 1.063 MJ m^−3^. To form the interlocked hybrid, the hydrogel precursor is drop cast onto the microfoam-attached substrate. An anionic surfactant has been added to the standard precursor for improved wettability, facilitating full infiltration into the porous microfoam. A cross-sectional optical image in Fig. 2c reveals a trilayer structure of the elastomer-hydrogel hybrid, with the interpenetrating hydrogel and SEBS elastomer as the intermediate bonding layer. Additionally, the SEM images in Fig. S5 further confirm that the hydrogel has completely infiltrated the porous microfoam. This ideal microstructure is attributed to the capillary action of the porous microfoam that facilitates the absorption of the hydrogel precursor.

The interlocked hybrid retains structural stability under large tensile deformations. The hydrogel is not peeled off from SEBS at 300% strain (Fig. 2d). The standard 180°-peeling test is carried out to evaluate the robustness of hydrogel–elastomer hybrids. Stiff backing layers are laminated on hydrogels and elastomers to constrain their elongations [46]. Directly stacked hydrogel–SEBS film hybrids show obvious interfacial crack propagation (Fig. 2e). A steady peeling force is measured as 2.5 N m^−1^, corresponding to an interfacial toughness of 5.6 J m^−2^ (Fig. 2f). In contrast, the hydrogel of the interlocked hybrid produces a brushed hair pattern near the interpenetrating bonding layer, as the characteristic signature of cohesive failures [46, 57]. The peeling force initially increases and approaches a stable value of ~ 78 N m^−1^. The interfacial toughness of 158.2 J m^−2^ represents a marked improvement over stacked hybrids by ~ 28 times. Instead of simply increasing the interface roughness, the hydrogel has been infiltrated into the open pores of the microfoams. This unique topological feature ensures the separation through rupture, leading to substantially enhanced interfacial interactions [52]. As the hydrogel has significantly lower toughness than the elastomer, the crack interface is expected to be confined within the hydrogel and has been confirmed in Supporting Video S1. According to SEM images in Fig. S6, the residual surface of the interlocked hybrid after peeling off the hydrogel layer reveals intact microfoam partially filled with the hydrogel. As a result, the microfoam establishes an interpenetrating interface with the hydrogel to achieve strong adhesion. On the other hand, the interfacial toughness value may not necessarily be the highest among recent reports. The PDA-PAM hydrogel used here is well recognized for its tissue affinity rather than its mechanical properties. Alternative toughened hydrogels can be used to augment the adhesion levels.

We have further investigated the influence of the microfoam on the mechanical interlocking effect. The porosity of the microfoam increases by raising the SA microrod loading, as shown in Fig. S7. The pore size remains relatively consistent despite some aggregations at high SA microrod loading. According to Figs. 2g and S8, the softness of the microfoam improves with its porosity, as reflected in reduced modulus and increased fracture strain. The robustness of the corresponding interlocked hybrids is characterized by 180°-peeling tests, as summarized in Fig. 2h. The interfacial toughness shows a slight initial increase with porosity before declining significantly at high porosity. A weight ratio of 4:1 between SA microrods and SEBS is found to be the optimal composition to attain strong adhesion. In addition, the interfacial toughness also depends on the microfoam thickness, as depicted in Fig. 2i. A thin microfoam has weak adhesion, likely associated with its soft mechanical properties. A decent thickness of 140 µm is still needed for robust attachments.

### Generalization to Other Polymers

**Fig. 3:**
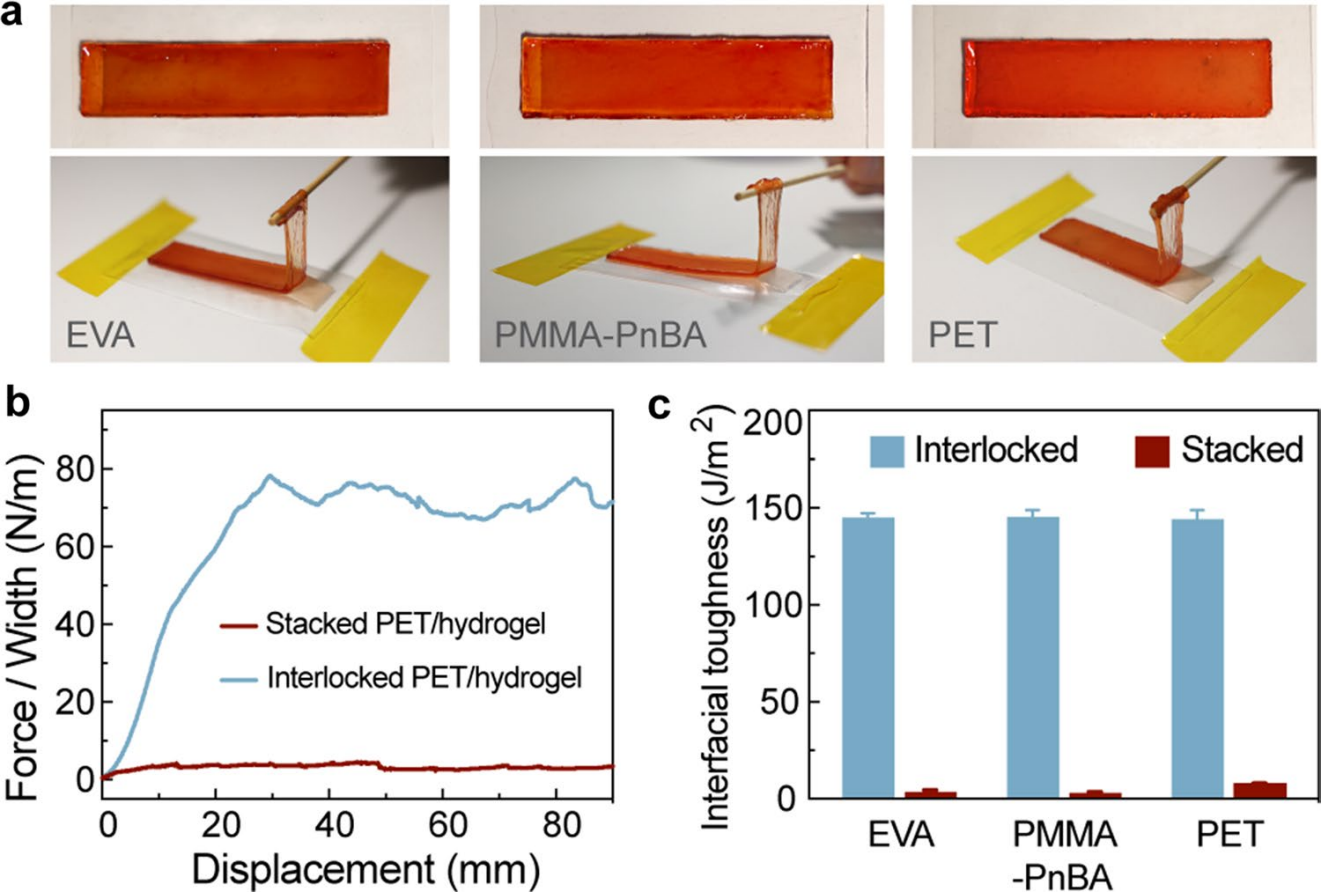
Interlocking hybrids on different substrates. **a** Optical images showing interlocking hybrids and corresponding manual peeling processes on EVA (left), PMMA-PnBA (middle), and PET (right) substrates. **b** Measured forces *versus* displacement during 180°-peeling tests for regular hydrogel-PET film hybrid and interlocked hydrogel-PET hybrid. **c** Interfacial toughness of three regular hybrids and corresponding interlocked hybrids

Bonding hydrogels to different polymer substrates is a widely recognized challenge. Here, the mechanical interlock utilizes the microfoam to form an interpenetrating interface with the hydrogel. To expand the range of compatible polymers, a petroleum resin is mixed with the SEBS elastomer during microfoam preparation to act as the tackifier [58]. The resulting microfoam can be easily laminated to various substrates through thermal bonding, including EVA, PMMA-PnBA elastomers, and PET plastics. According to Fig. S9, the interfacial toughness for EVA, PMMA-PnBA, and PET is 490.8, 660.6, and 195.2 J m^−2^, respectively. As shown in Fig. 3a, these microfoams allow the facile preparation of interlocked hydrogel/polymer hybrids, in which the hydrogels are firmly adhered to these polymers. Figure 3b shows the 180°-peeling tests on PET/hydrogel hybrids. The peeling force is ~ 75 N m^−1^ for the interlocked hybrid and ~ 3.6 N m^−1^ for the regularly stacked hybrid. In Fig. 3c, the interfacial toughness of the three interlocked hybrids is quite high and consistent with the interlocked hydrogel/SEBS hybrid. This mechanical interlocking strategy has shown significant improvements over regularly stacked hybrids, demonstrating its generic suitability for different substrates.

In addition to SEBS-based elastomers, the material choice for the microfoams can be replaced with other polymers. For instance, we have prepared EVA microfoams using a similar sacrificial template approach. These EVA microfoams have the ability to bond with EVA and SEBS substrates, consequently creating interlocked elastomer/hydrogel hybrids, as shown in Fig. S10. The results suggest the soft microfoam as a versatile intermediate layer that can be used for rugged integration with hydrogels.

### Electrical Properties and Body Conformability of Interlocked Hybrid Electrodes

**Fig. 4:**
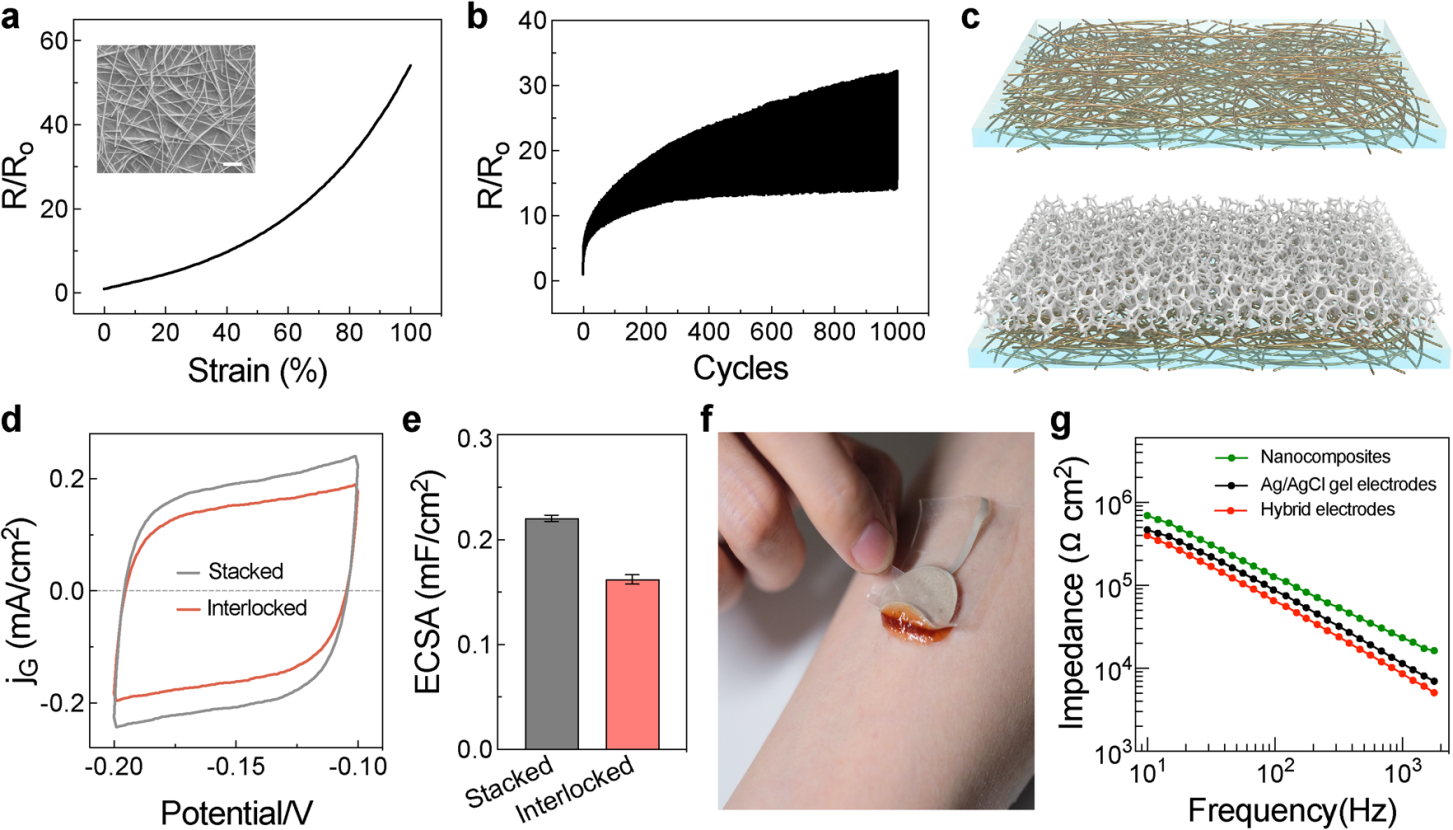
Electrical properties of interlocked hybrid electrodes. **a** Normalized resistance of the Ag NW nanocomposite as a function of the tensile strain. Inset: SEM image showing randomly oriented Ag NWs embedded in the SEBS elastomer matrix. Scale bars: 2 μm. **b** Change in the resistance of Ag NW nanocomposites during 1000 stretch–relaxation cycles to 50% strain. **c** Schematic diagram illustrating the pristine (left) and microfoam-attached (right) Ag NW nanocomposites. **d** Cyclic voltammetry curves of bare and microfoam-attached nanocomposite electrodes at a scan rate of 0.9 V s^−1^. **e** Corresponding double-layer capacitance values. **f** Optical image shows a representative hybrid electrode firmly attached to the skin. **g** Skin–electrode contact impedance as a function of frequency for epidermal electrodes. Commercial Ag/AgCl gel electrodes establish the baseline for comparison. Ag NW nanocomposite electrodes are applied with 4 kPa pressure through a compression wrap for reliable skin contact

Ag NW nanocomposites are a prototypical form of compliant conductors [16, 18]. In the inset of Fig. 4a, SEM image reveals randomly oriented Ag NWs in the SEBS elastomer matrix. The percolation network of high–aspect–ratio Ag NWs is responsible for the excellent electrical conductivity of ~ 11,000 S cm^−1^. Abundant Ag NWs are exposed on nanocomposite for convenient contact with other conductors. The stretchability of Ag NW nanocomposites is evaluated by measuring their electrical properties under uniaxial tensile deformations, as shown in Fig. 4a. The normalized resistance is 4.74 at 20% strain, 13.53 at 50% strain, and 54.08 at 100% strain, respectively. The decent deformability is a notable attribute of nanowire percolation networks [16, 17]. In Fig. 4b, the Ag NW nanocomposites exhibit limited resistance changes during 1000 stretch-relaxation cycles to 50% strain, which demonstrates excellent electromechanical durability for wearable applications. Unlike continuous metal films, the nanocomposite relies on the exposed Ag NWs for external contact, as illustrated in Fig. 4c. To estimate the actual surface area, the double-layer capacitance of the nanocomposite electrode submerged in PBS electrolyte is measured with cyclic voltammetry (Fig. 4d) [59, 60]. The capacitance of the pristine Ag NW nanocomposite is determined as 0.22 mF cm^−2^ (Figs. 4e and S11). After microfoam attachment, the capacitance shows a certain decrease to 0.16 mF cm^−2^, suggesting a slightly reduced surface area by 27.3%. The nanocomposite electrode is still largely exposed for reliable contact with the hydrogel, thanks to the highly porous structure of the microfoam (Fig. 2b).

Interlocked nanocomposite/hydrogel hybrid electrodes are created for epidermal sensing. The attached microfoam and hydrogel have minor influences on the electrode durability, as confirmed by the tensile fatigue test presented in Fig. S12. In Fig. 4f, we can observe that the interlocked electrode has achieved firm attachment to the skin. According to 180°-peeling tests conducted on porcine skin, the interfacial adhesion toughness of our PDA–PAM hydrogel is determined as ~ 20 J m^−2^ (Fig. S13). This excellent tissue adhesion of the hydrogel is consistent with previous studies due to the abundant catechol groups with high cellular affinity [61]. The mechanically interlocked interface further allows the convenient removal of electrodes without accidental delamination. In hybrid electrodes, Ag NW nanocomposites and hydrogels are also electrically connected through the porous locking layer. Figure 4g shows the skin–electrode contact impedance as a function of the frequency. The hybrid electrodes exhibit lower contact impedance than commercial Ag/AgCl gel electrodes. Since the conformability of epidermal electrodes is directly correlated with their bending stiffness [23, 62], the hydrogels have a much lower modulus than regular elastomers, allowing them to make better contact with the textured skin surface through deformations. The exceptional contact impedance of the interlocked hybrid electrodes is attributed to the high ionic conductivity, low modulus, and strong tissue affinity of the PDA-PAM hydrogel layer to interface with the skin. On the other hand, traditional nanocomposite electrodes cannot be directly mounted on the curvilinear human body. An applied pressure of 4 kPa is required to compress the Ag NW nanocomposite electrode onto the textured skin surface. However, the contact impedance is still significantly inferior to the hybrid electrodes. The ordering of the contact impedance has been confirmed by additional measurements from five individuals, as summarized in Fig. S14. Therefore, the interlocked nanocomposite/hydrogel hybrid electrodes are considered an attractive mechanical and electrical interface for epidermal electronics.

### Epidermal Biopotential Detection

**Fig. 5:**
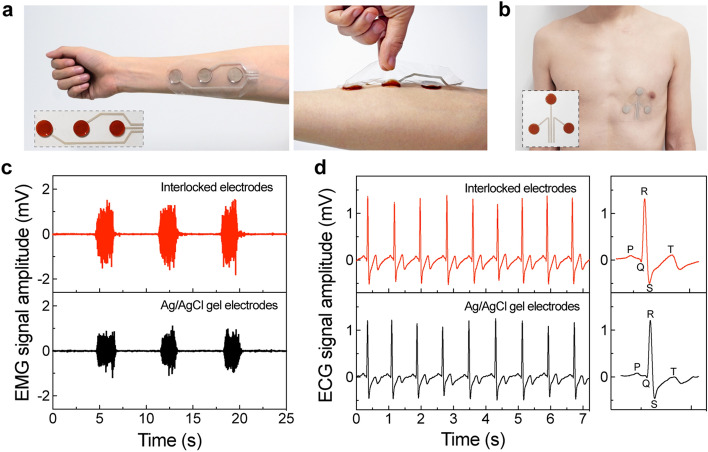
Interlocked hybrid electrodes for biopotential recordings. **a** Images of an epidermal sensing patch fixed on the forearm to record EMG signals. Inset: an epidermal EMG sensing patch comprised of three sensing electrodes and interconnects. **b** Optical image showing an epidermal patch mounted on the left chest to record ECG signals. Inset: an epidermal ECG sensing patch. **c** Recorded EMG waveforms with interlocked hybrid electrodes (top) and commercial Ag/AgCl gel electrodes (bottom) corresponding to several muscle contraction-relaxation cycles. **d** Recorded ECG signals (left) exhibiting characteristic P, Q, R, S, and T signatures (right)

Encouraged by the above results, we construct epidermal electronic patches with interlocked hybrid electrodes for biopotential recordings. A typical patch is composed of three interlocked electrodes and corresponding interconnects (Fig. 5a). An epidermal sensing patch with linearly arranged electrodes was placed on the right flexor carpi radialis for EMG recording. The hydrogel electrodes exhibit firm adhesion to the skin due to polydopamine. Likewise, a sensing patch with triangularly arranged electrodes was attached to the left chest for ECG recording (Fig. 5b). Raw biopotentials were conditioned with a preamplifier and sampled with a data acquisition card. In Fig. 5c, the EMG signals reveal the contraction of muscle fibers corresponding to open and closed hand motions. The signal-to-noise ratio (SNR) is 29.0 dB for interlocking electrodes and 26.2 dB for commercial gel electrodes. In addition, ECG waveforms reveal clear P, Q, R, S, and T signatures critical for the diagnosis of cardiovascular diseases, as shown in Fig. 5d [63, 64]. The SNR by interlocking electrodes (38.7 dB) is also higher than that by commercial gel electrodes (33.9 dB). The improved signal quality of interlocking electrodes is consistent with their low skin contact impedance. In addition, the self-adhesive hydrogel electrodes can be painlessly peeled off from the skin due to their strong adhesion to the underlying substrate. The as-prepared conductive composites are therefore well suited for robust epidermal electronics.

### Fabrication and Application of Integrated Epidermal Sensing Systems

**Fig. 6:**
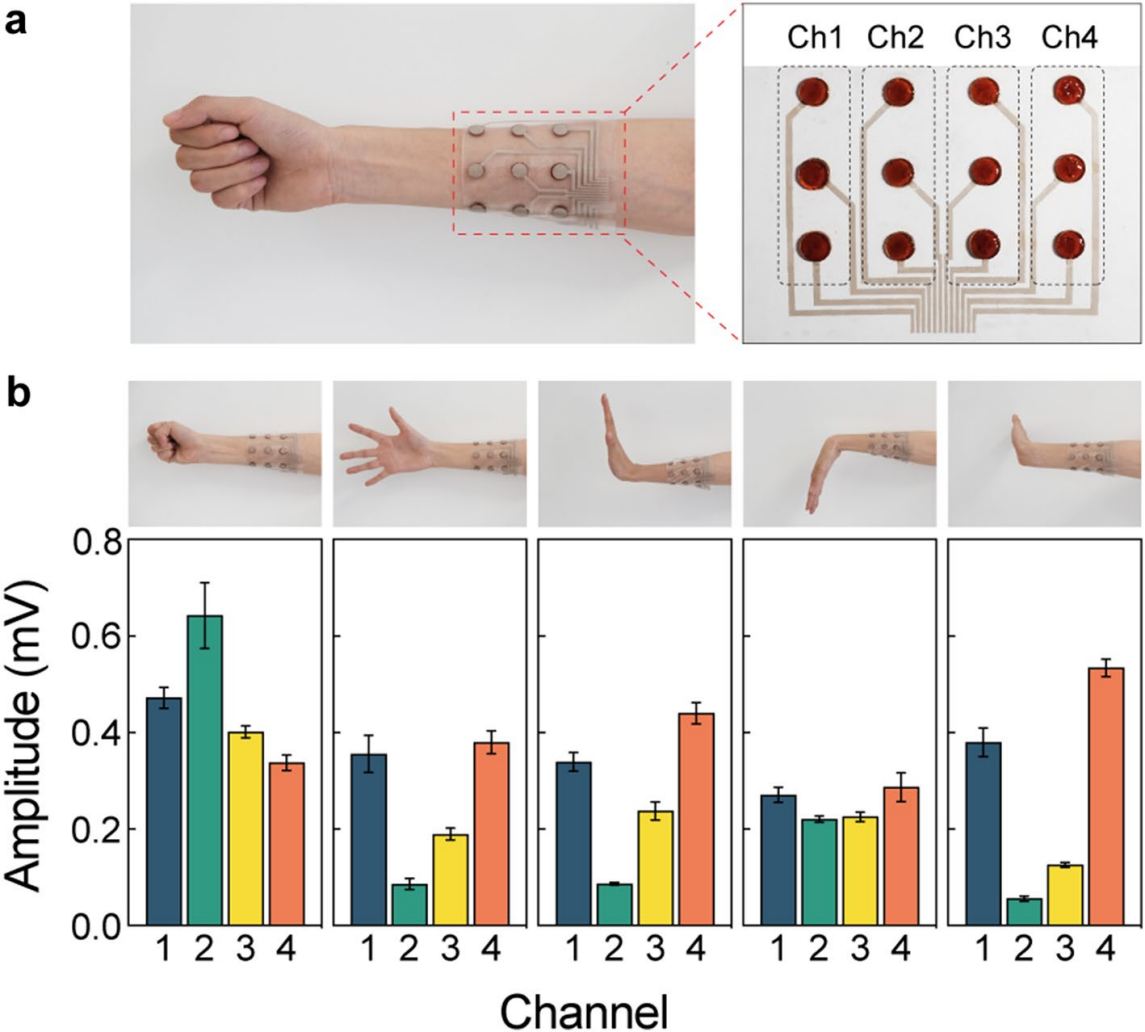
Integrated epidermal electronic sleeve as a human–machine interface. **a** Optical images showing an epidermal electronic sleeve wrapping over the forearm (left) and the integrated four-channel EMG sensing system (right). **b** Voltage amplitude of each sensing channels (bottom) for different hand gestures (top)

An integrated epidermal sensing system is fabricated as a human–machine interface. Figure 6a shows the integrated sensing system as a soft armband composed of twelve sensing electrodes. Each set of three electrodes makes up an independent EMG channel designed to monitor the activity of an individual muscle. The sensing electrodes and electrical interconnects are spatially separated to avoid crosstalk among the channels. Individual sensing channels are strategically positioned along the primary muscles of the forearm, including brachioradialis, flexor carpi radialis, flexor carpi ulnaris, and extensor digitorum. The self-adhesive hydrogel electrodes exhibit conformal and intimate attachment to the curvilinear human body, establishing a stable interface for high-fidelity electrophysiological recording. In Fig. S15, the conditioned EMG waveforms are simultaneously recorded for different hand gestures. Common hand gestures are readily identified with root-mean-square voltage amplitudes reflecting muscle contraction levels, as shown in Fig. 6b. The entire system essentially represents an epidermal human–machine interface to decipher the gestures into four channels of analog signals, which is attractive for robotic control, advanced prosthetics, and virtual reality.

## Conclusions

In conclusion, we have presented a convenient fabrication approach for conductive nanocomposite/hydrogel hybrid electrodes with a mechanically interlocked design. Soft microfoams thermally bonded to the conductive nanocomposite establish an interpenetrating network with the hydrogel. These interlocked hybrids exhibit a high interfacial toughness of 158.2 J m^−2^, surpassing the directly stacked hybrid by 28 times. Ag NW nanocomposites and hydrogels are found to be electrically connected through the porous microfoams. Interlocked hybrid exploits PDA-PAM hydrogel to establish a conformal and self-adhesive interface with the skin, achieving lower contact impedance than commercial Ag/AgCl gel electrodes. These properties make them attractive epidermal electrodes to capture high-quality EMG and ECG signals. An integrated epidermal sleeve is created as a human–machine interface to distinguish hand gestures through recorded muscle contractions. The microfoam-enabled bonding strategy is generally applicable to various substrates, including EVA, PMMA-PnBA, and PET, and different microfoam materials. The mechanically interlocked hybrid electrodes merge the attributes of nanocomposites and hydrogels, making them attractive candidates for emerging applications in epidermal devices and systems.

## Supplementary Information

Below is the link to the electronic supplementary material.

Supplementary file1 (MOV 13137 kb)

Supplementary file2 (PDF 1123 kb)
