# Supplementary material for: A Generic Strategy to Create Mechanically Interlocked Nanocomposite/Hydrogel Hybrid Electrodes for Epidermal Electronics
Source: Nanomicro Lett. 2024 Jan 12;16:87. doi: 10.1007/s40820-023-01314-z (PMC10786775; doi:10.1007/s40820-023-01314-z)
Supplement: Supplementary file 2 — Supplementary file2 (PDF 1123 kb) [file 40820_2023_1314_MOESM2_ESM.pdf]

Supporting Information for

# A Generic Strategy to Create Mechanically Interlocked Nanocomposite/Hydrogel Hybrid Electrodes for Epidermal Electronics

Qian Wang[1,2], Yanyan Li[1,2], Yong Lin[1,2], Yuping Sun[1,2], Chong Bai[1,2], Haorun Guo[3], Ting Fang[1,2], Gaohua Hu[1,2], Yanqing Lu[1,4,]*, and Desheng Kong[1,2,]*

[1] College of Engineering and Applied Sciences, National Laboratory of Solid State Microstructure, and Collaborative Innovation Center of Advanced Microstructures, Nanjing University, Nanjing 210023, P. R. China

[2] State Key Laboratory of Analytical Chemistry for Life Science, and Jiangsu Key Laboratory of Artificial Functional Materials, Nanjing University, Nanjing 210023, P. R. China

[3] College of Chemical Engineering and Technology, Engineering Research Center of Seawater Utilization Technology of Ministry of Education, State Key Laboratory of Reliability and Intelligence of Electrical Equipment, Hebei University of Technology, Tianjin 300130, P. R. China

[4] Key Laboratory of Intelligent Optical Sensing and Manipulation, Nanjing University, Nanjing 210093, P. R. China

*Corresponding authors. E-mail: dskong@nju.edu.cn (D. Kong); yqlu@nju.edu.cn (Y. Lu)

## Supplementary Figures

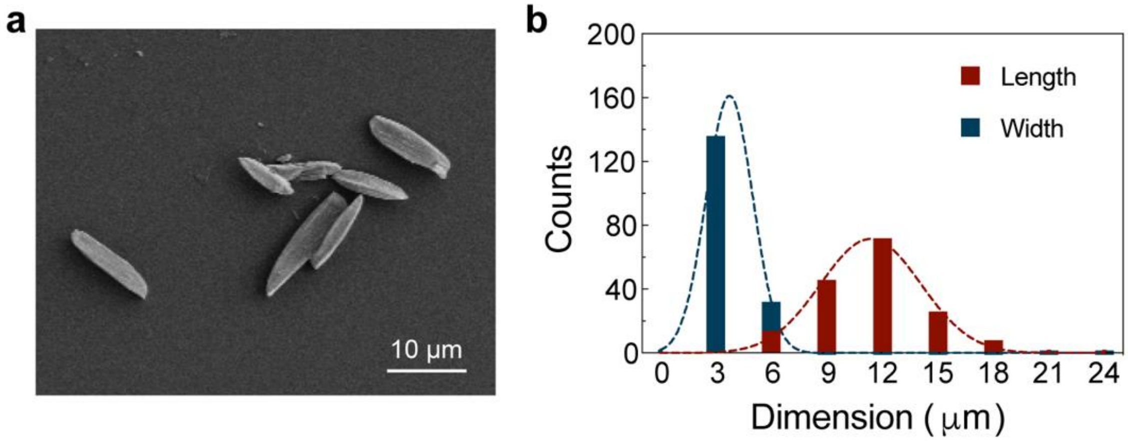


**Fig. S1** (**a**) SEM image showing the microstructures of salicylic acid microrods. (**b**) Histograms of length and width distributions. Gaussian fits to the distributions (smooth curves) yield 11.4 ± 2.7 μm for the length and 3.7 ± 1.3 μm for the width

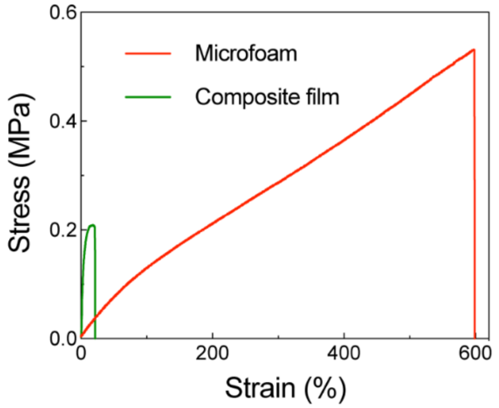


**Fig. S2** Uniaxial stress–strain curves of SEBS/salicylic acid composite and the corresponding microfoam

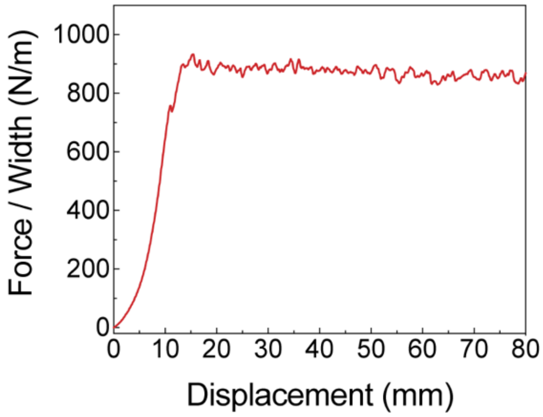


**Fig. S3** 180°-peeling force *versus* displacement for SEBS microfoam thermally laminated on SEBS substrate. The corresponding interfacial toughness is 1776.5 J $m^{-2}$

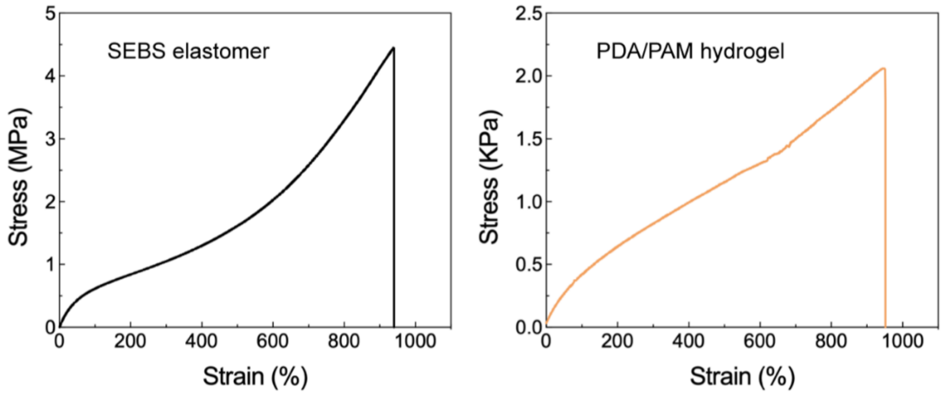


**Fig. S4** Uniaxial stress–strain curve of the SEBS elastomer (left) and PDA/PAM hydrogel (right). The SEBS elastomer has a modulus of 1.3 MPa, a fracture strain of 941%, and a toughness of 1716 MJ $m^{-3}$. The hydrogel has a modulus of 0.54 KPa, a fracture strain of 952%, and a toughness of 1.063 MJ $m^{-3}$

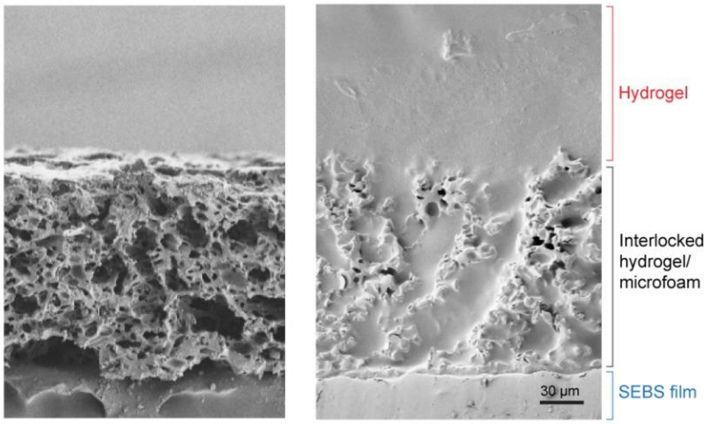


**Fig. S5** Cross-sectional SEM images of microfoam-attached SEBS film (left) and mechanically interlocked hydrogel/microfoam hybrid (right)

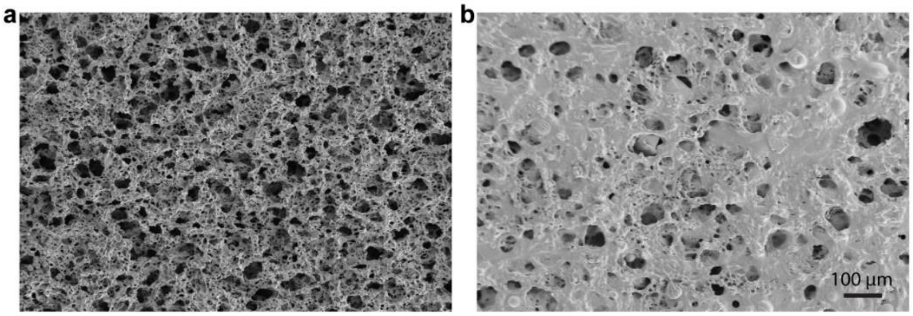


**Fig. S6** (**a**) SEM image of the SEBS microfoam attached to the SEBS substrate. (**b**) SEM image showing the residual surface of the interlocked hybrid after peeling off the hydrogel layer. The microfoam remains on the substrate and is partially filled with the hydrogel

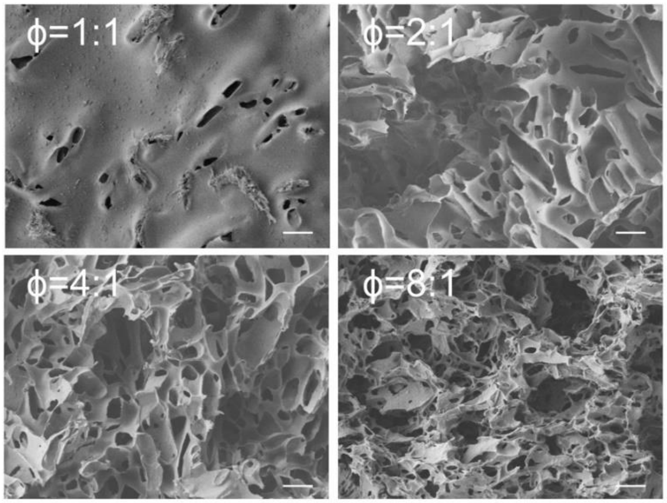


**Fig. S7** SEM image showing SEBS microfoams with different porosity. In the sacrificial template synthesis, the porosity is modulated by the weight ratio (ϕ) between salicylic acid and SEBS. Scale bars: 10 μm

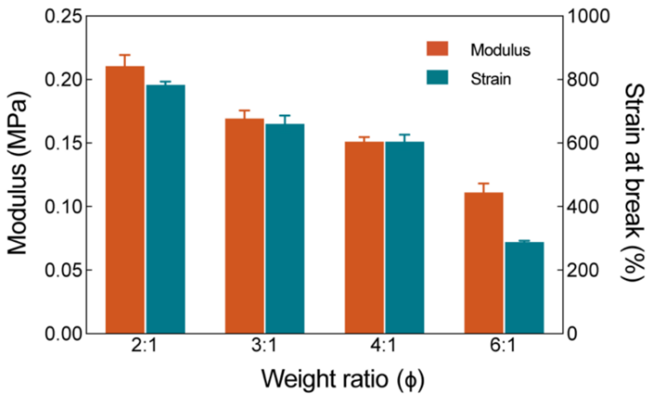


**Fig. S8** Young's Modulus and fracture strain of SEBS sponge with different porosity. The porosity is modulated by the weight ratio (ϕ) between salicylic acid and SEBS elastomer

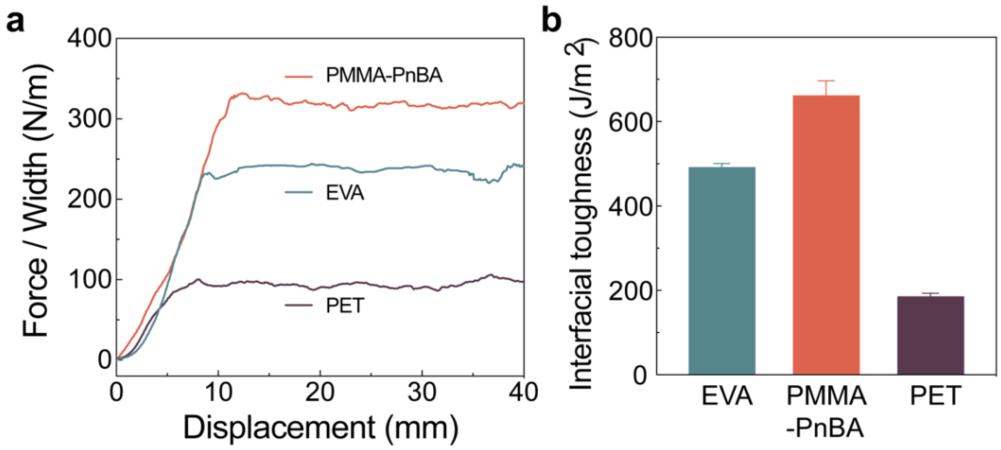


**Fig. S9** (**a**) 180°-peeling force *versus* displacement for tackified microfoam bonded to different substrates, including EVA, PMMA-PnBA, and PET. (**b**) Corresponding interfacial toughness

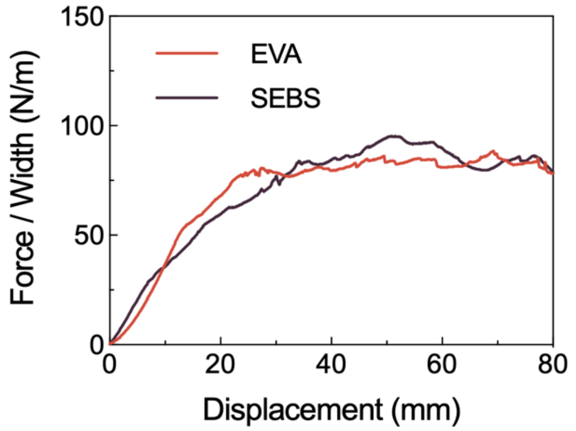


**Fig. S10** 180°-peeling force *versus* displacement curves for interlocked EVAmicrofoam/hydrogel hybrids on EVA and SEBS substrates

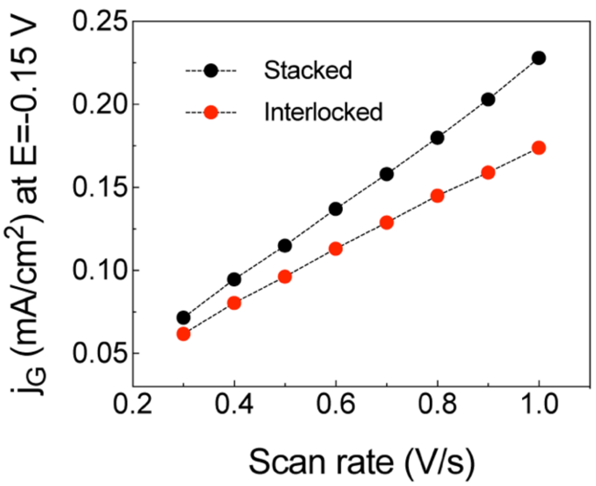


**Fig. S11** Capacitive current *versus* scan rate of Ag NW nanocomposite electrodes with/without microfoam attachment

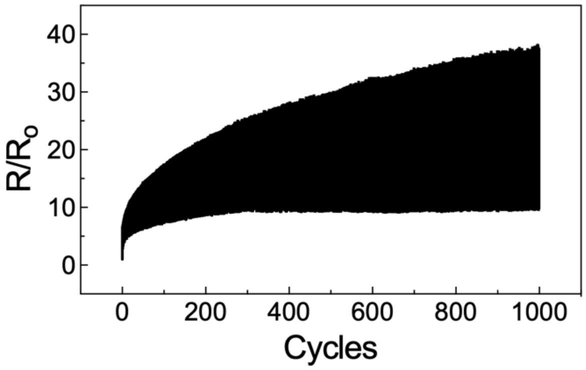


**Fig. S12** Change in the resistance of the hybrid electrode during 1000 stretch–relaxation cycles to 50% strain

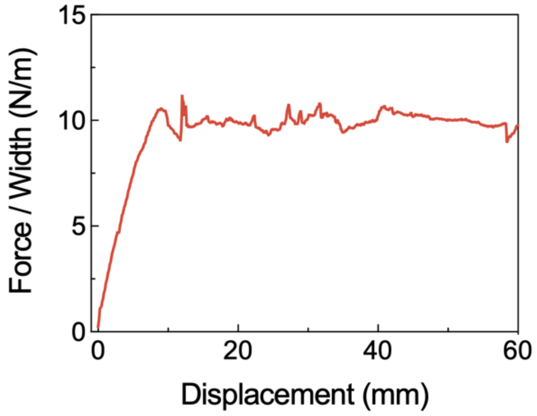


**Fig. S13** 180°-peeling force *versus* displacement curves for PDA-PAM hydrogel on porcine skin. The interfacial adhesion toughness is determined as ~20 J $m^{-2}$

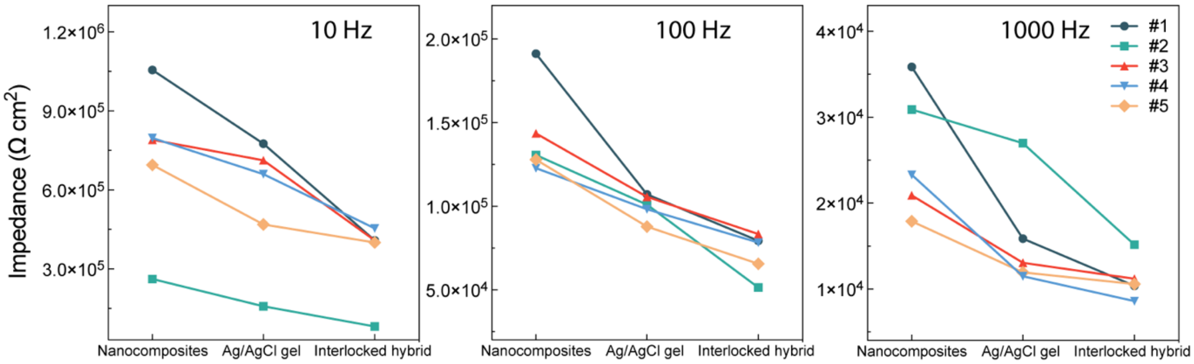


**Fig. S14** Skin-electrode contact impedance from multiple human subjects at selected frequencies of 10 (left), 100 (middle), and 1000 (right) Hz. The contact impedance decreases in the order of nanocomposite, Ag/AgCl gel, and interlocked hybrid electrodes

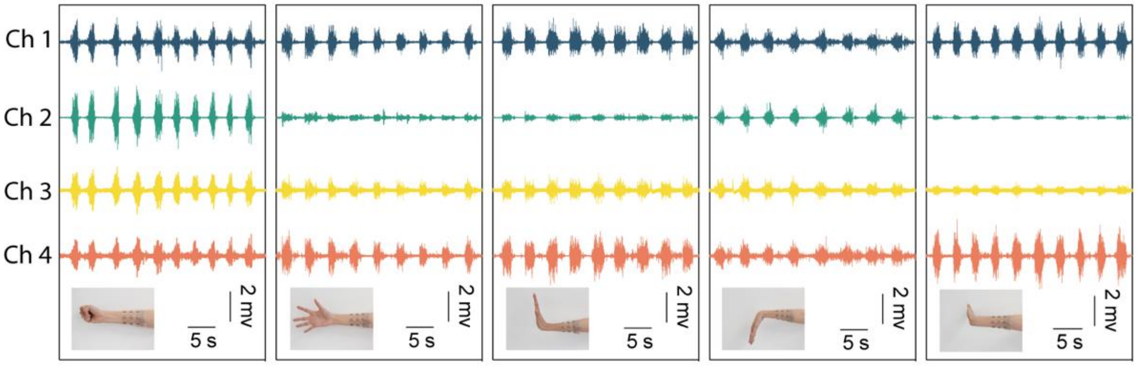


**Fig. S15** Four-channel EMG waveforms acquired from the epidermal sensing sleeve in response to different hand gestures

## Supplementary Video

**Video S1** Dynamic peeling process of the hydrogel layer from the interlocked hybrid revealed by optical microscopy
